# Reasons for surgical revision after conservatively treated radial head fractures—retrospective study of 70 patients

**DOI:** 10.1007/s11678-018-0456-2

**Published:** 2018-05-15

**Authors:** R. Nietschke, K. J. Burkhart, B. Hollinger, F. I. Dehlinger, A. Zimmerer, M. M. Schneider

**Affiliations:** 1 Upper Extremity Unit, ARCUS Sportklinik, Rastatter Str. 17–19, 75179 Pforzheim, Germany; 2 University Witten/Herdecke, Witten, Germany; 3 ACURA Kliniken, Albstadt, Germany

**Keywords:** Radius fractures, Elbow, Joint instability, Osteoarthritis, Surgery, Radiusfrakturen, Ellenbogen, Gelenkinstabilität, Ellenbogensteife, Operation

## Abstract

**Background:**

An inadequate clinical outcome after conservatively treated radial head fractures is not uncommon. We analyzed the subjective limitations, objective complaints, and surgical procedures for radial head fractures initially treated conservatively.

**Patients and method:**

Between 2007 and 2016, 70 patients (42 men, 28 women) who suffered from fracture sequelae after conservatively treated radial head fractures were examined. Demographic (age, 41.8 years, range, 16–75 years) and clinical data (pain, range of motion, instability) were retrospectively evaluated.

**Results:**

The average time to surgery after trauma was 50 months (range, 5–360 months). In 38 cases, radial head fractures were initially treated with immobilization for 3.4 weeks (range, 1–8 weeks). Physiotherapeutic treatment was performed in 39 cases. In only half of the cases was retrospective Mason classification possible: 20 type I, 8 type II, 5 type III, and 2 type IV. Of the 70 patients, 53 had posttraumatic elbow stiffness; 34 had isolated lateral and four patients isolated medial ligament instability. There were eight cases with a combination of lateral and medial ligament instability and 27 cases of elbow stiffness combined with instability. An average of 1.2 (range, 1–4) surgical procedures per patient were performed. In all, 64 patients underwent elbow arthroscopy with arthrolysis and additional treatment depending on other injuries. The range of motion improved on average from preoperative flexion/extension of 131–15–0° to postoperative flexion/extension of 135–5–0° (gain in flexion: 4.2° and extension: 10.6°).

**Conclusion:**

Conservative treatment of radial head fractures does not always yield good results. Reasons for a poor outcome include chronic instability, cartilage damage, stiffness, or a combination thereof. Improved outcomes can be achieved via arthroscopic arthrolysis.

## Zusammenfassung

**Hintergrund:**

Patienten mit unzureichendem klinischem Ergebnis nach konservativ behandelten Radiuskopffrakturen sind nicht selten. Ziel der vorliegenden Studie war eine Analyse von subjektiven Einschränkungen, objektiven Beschwerden und chirurgischen Eingriffen nach anfänglich konservativ behandelten Radiuskopffrakturen.

**Patienten und Methode:**

Zwischen 2007 und 2016 wurden 70 Patienten (42 Männer, 28 Frauen) untersucht, die an Frakturfolgen nach konservativ behandelten Radiuskopffrakturen litten. Demographische (Alter im Mittel: 41,8 Jahre; Spanne: 16–75 Jahre) und klinische Daten (einschließlich Schmerzen, Bewegungsumfang, Instabilität) wurden retrospektiv ausgewertet.

**Ergebnisse:**

Im Durchschnitt betrug die Zeit nach dem Trauma 50 (Spanne: 5–360) Monate. In 38 Fällen wurden Radiuskopffrakturen zunächst für 3,4 (Spanne: 1–8) Wochen mittels Immobilisation behandelt. Eine physiotherapeutische Behandlung wurde in 39 Fällen durchgeführt. In nur der Hälfte der Fälle war eine retrospektive Klassifizierung nach der Mason-Klassifikation möglich: 20 Typ I, 8 Typ II, 5 Typ III und 2 Typ IV. Bei 53 der 70 Patienten lag eine posttraumatische Ellenbogensteife vor, bei 34 eine isolierte laterale und bei 4 Patienten eine isolierte mediale Seitenbandinstabilität. Eine Kombination aus lateraler und medialer Seitenbandinstabilität bestand in 8 Fällen, Ellenbogensteife kombiniert mit einer Instabilität in 27 Fällen. Pro Person waren 1,2 (Spanne: 1–4) chirurgische Eingriffe notwendig. Bei 64 Patienten wurden jeweils eine Arthroskopie (52-mal kombiniert mit einer Arthrolyse) und weitere notwendige Behandlungen je nach Begleitverletzungen durchgeführt. Der Bewegungsumfang verbesserte sich im Durchschnitt von Flexion/Extension (präoperativ) 131–15–0° zu Flexion/Extension (postoperativ) 135–5–0° (Zugewinn: Flexion: 4,2°; Extension: 10,6°).

**Schlussfolgerung:**

Die konservative Behandlung von Radiuskopffrakturen zeigt nicht immer gute Ergebnisse. Gründe hierfür können chronische Instabilitäten, Knorpelschäden, Steife oder eine Kombination davon sein. Eine Verbesserung des Ergebnisses kann über eine arthroskopische Arthrolyse erzielt werden.

More than 30% of all fractures are radial head fractures, making them the most common bony injuries of the elbow joint. They account for approximately 1.5–4% of all fractures in the human body [21].

**Fig. 1:**
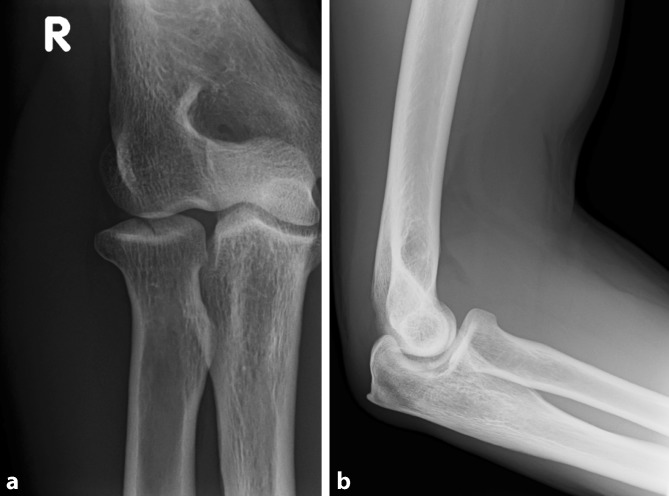
Radiographs (two planes) of a radial head fracture (Mason I)

**Fig. 2:**
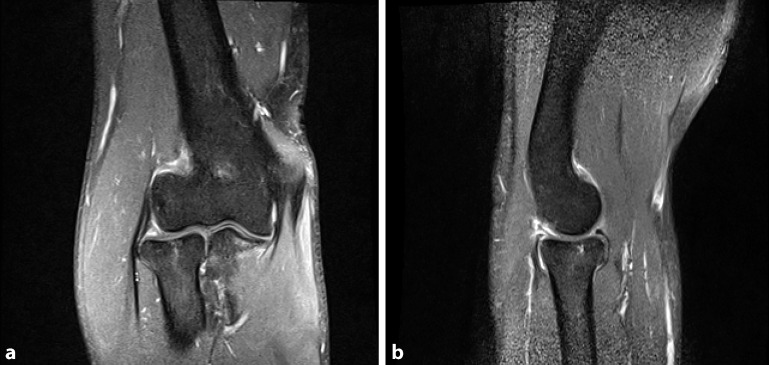
Magnetic resonance imaging of Mason I radial head fracture and absence of soft tissue damage

**Fig. 3:**
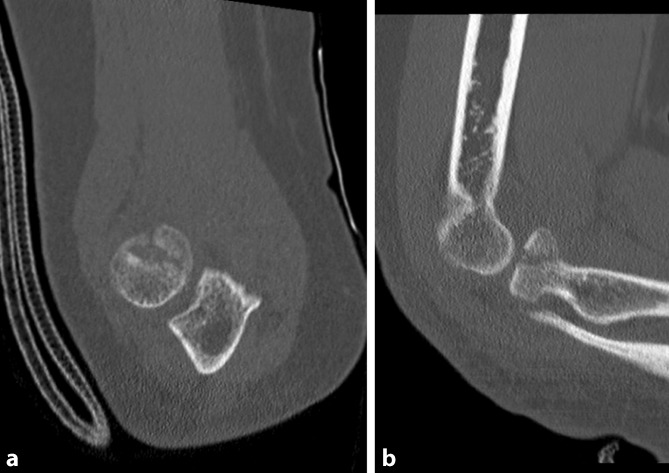
Computed tomography scans of a radial head fracture (Mason II)

The classification according to Mason [31], later modified according to Johnston, is the most commonly used classification system for radial head fractures (Figs. 1, 2 and 3).

The frequencies of the different grades were published in 2010 and 2011. Mason I fractures accounted for about two thirds of all fractures, while only 2.5% were Mason IV fractures [11, 23].

Since Mason I fractures are generally regarded as harmless injuries, conservative therapy is the method of choice ([6, 11, 17, 27, 31]; Figs. 1 and 2). Conservative therapy includes immobilization for 5–7 days followed by early functional treatment [8, 29, 30, 33, 41, 42]. The outcomes after conservative treatment are good to very good in about 90% of cases [17, 43, 45]. Only few data exist concerning trauma sequelae of radial head fractures that led to poor results. Burkhart et al. reported on a case series of 16 patients with poor clinical results following Mason I fractures. In their study, 62.5% of patients had symptomatic posttraumatic osteoarthritis with elbow stiffness and free joint bodies. Five of these patients had a relevant posterolateral rotation instability or a bilateral instability, which had to be addressed by ligament reconstruction. One patient developed a symptomatic hypertrophic plica [6]. The authors concluded that the trauma mechanism is nearly the same as in elbow dislocations. In some cases, there was evidence on magnetic resonance imaging (MRI) or intraoperative findings that the Mason I fracture was actually a Mason IV fracture. In addition, Davidson et al. demonstrated, as part of stress tests with radial head fractures, that all fractures had instabilities due to either valgus or axial stress [9]. Itamura et al. emphasized that only 12.5% of the radial head fractures in their case series did not have any relevant ligament injuries [19].

In the case of Mason II fractures, good results are achieved by conservative and operative measurements alike [2, 10, 15, 18, 28].

Lindenhovius and coworkers did not find better clinical long-term results following operative treatment of Mason II fractures in comparison with the long-term results of nonoperative management published by Akesson et al. [2, 28]. The high rate of posttraumatic arthritis in the nonoperative group in the latter study is noteworthy. Although the authors state that these cases are asymptomatic, we believe this to be a worrisome aspect of the conservative treatment of displaced radial head fractures as radiocapitellar arthritis is known to be one of the most challenging problems in elbow surgery—especially in the young and active patient. Therefore, the present study evaluates and describes the sequelae of failed conservative treatment after radial head fractures.

## Patients and methods

### Patients

This retrospective, descriptive, and explorative observational study included patients who underwent surgery for fracture sequelae after conservatively treated radial head fractures. We identified 70 patients (28 women and 42 men) treated between 2007 and 2016. Initial treatment was conducted outside our hospital. Patients were referred to our elbow center after their initial presentation elsewhere. The average age of the patients was 41.83 years (range, 16–75 years). There were 40 right and 30 left elbows affected.

On average, the duration of conservative therapy was 50 months (range, 5–360 months) from the time of trauma to surgery.

The study included all patients who underwent conservative treatment for at least 5 months or longer. We also included eight patients (11.4%) who had previous surgeries, e.g., arthrolysis, elsewhere.

### Arthroscopy

The procedure for arthroscopy is described here. We created five standard working portals: anteroradial, antero-ulnar, trans-tricipital, proximal dorsoradial, and distal dorsoradial.

After joint insufflation through the soft spot portal with 20 ml of normal saline solution, an inflow cannula was placed for the continuous supply of arthroscopy fluid via the anteroradial portal. The entire dorsal elbow joint section was inspected with the camera via the high dorsoradial portal and, if necessary, treated using the trans-tricipital portal. In addition, the camera was tilted laterally along the olecranon tip toward the deep dorsoradial portal.

Finally, we used the deep dorsoradial portal as an access to test the stability with an exchange rod inserted into the ulnohumeral joint.

We distinguished between three degrees of instability, according to the classification of O’Driscoll et al. [38]:

- Grade I: subluxation (posterolateral rotatory instability, PLRI)
- Grade II: incomplete dislocation (PLRI II)
- Grade III: complete dislocation (PLRI III)

The anterior joint compartment was examined by using the anterolateral portal. If necessary, arthroscopic cartilage debridement, micro-fracturing, synovectomy, and/or capsulectomy was carried out after creating an antero-ulnar portal.

Medical files were analyzed according to the fracture sequelae, range of motion, stability, and patient complaints. Each of our patients was re-evaluated 6 weeks postoperatively. Patient data were collected retrospectively and postoperative evaluation was completed with the help of the available medical history. No telephone interview or personal examination was performed in this study.

## Results

In 35 cases (50.0%), retrospective grading according to the Mason classification could not be made because radiological images at the time of the trauma were absent and there was incomplete or missing data on the initial classification. In the remaining 35 patients, there were 20 type I, eight type II, five type III, and two type IV fractures.

Approximately half of our patients (55.7%) were immediately immobilized with a plaster after trauma. The duration of the immobilization was 3.4 weeks (range, 1–8 weeks) on average. Five patients (7.1%) were treated with orthopedic devices after trauma (e.g., hinged external fixator, etc.).

In all, 98.6% of patients complained of pain in the affected elbow; only one patient specified no pain.

### Ligament instability and elbow stiffness

**Fig. 4:**
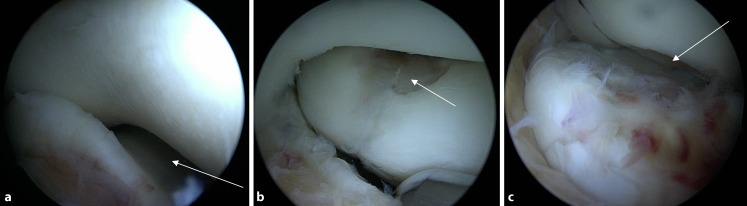
Gaping joint gap (*arrow in ***a**) during arthroscopy and advanced cartilage damage on the radial head and the capitulum humeri (*arrows in ***b**, **c**)

In 35 patients (50%), clinical examination revealed ulnar (*n* = 3), radial (*n* = 5), or bilateral lateral (*n* = 30) ligament instability of the elbow joint (Fig. 4a). A combination of elbow instability with concomitant posttraumatic elbow stiffness was found in 27 patients (38.6%).

**Fig. 5:**
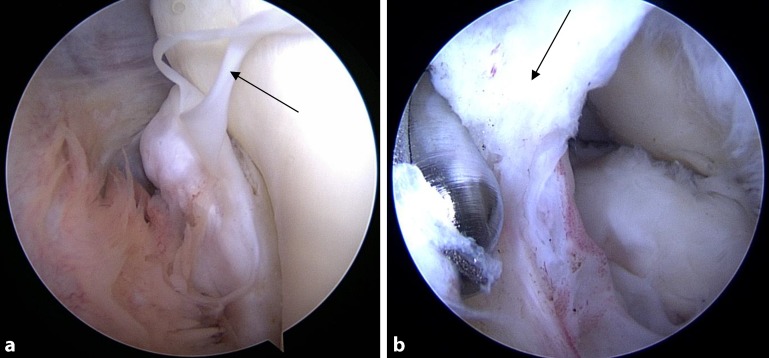
Distinct soft-parted bridle strands (*arrows*)

In all, 53 patients (75.7%) had symptomatic elbow stiffness with a restricted range of motion (Fig. 5a, b). Of these patients, 91% needed arthroscopic arthrolysis including capsulectomy. In addition to the arthroscopic arthrolysis, a total of two open arthrolysis procedures had to be performed. The preoperative range of motion was flexion/extension of 131–15–0° and pronation/supination of 67–0–71°. At the 6‑week follow-up, the range of motion was improved to flexion/extension of 135–5–0° as well as pronation/supination of 73–0–76°. Extension was improved by 10.6° and flexion by 4.2°, which corresponds to a total gain in range of motion of 14.8°.

Usually, the arthrolysis treatment was planned for cases of considerable elbow stiffness to improve range of motion and to prepare for a second ulnar or radial ligament reconstruction, if necessary.

Via arthroscopic stability testing with the exchange rod, 18 cases (25.7%) of PLRI II or more and 12 cases (17.1%) of ulnar instability were detected. There was bilateral lateral ligament instability in eight patients (11.4%). All the other patients had either no PLRI or had lateral instabilities of a grade less than PLRI II; 24 patients exhibited no instability at all (34.3%).

In five cases (7.1%), radial ligament reconstruction had to be carried out in a second surgery (either planned or due to recurrent instability).

**Fig. 6:**
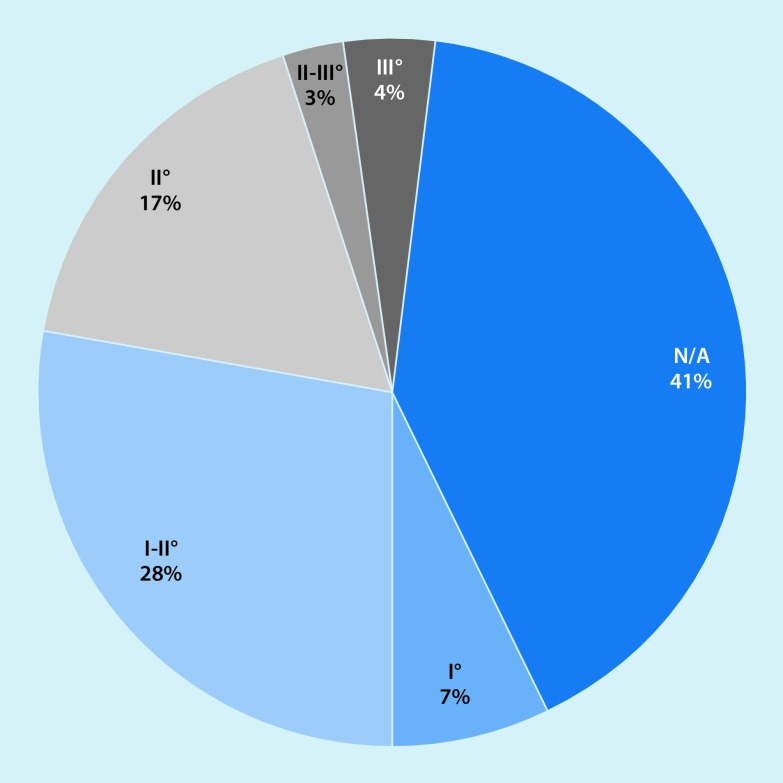
Distribution of posterolateral rotatory instability (*PLRI*) in the study series: 24% of the instabilities were graded as PLRI II or greater. *N/A* no PLRI

Four patients (5.7%) required either a radial (*n* = 2) or ulnar (*n* = 2) ligament reconstruction without previous arthroscopy (Fig. 6). In one case, ulnar ligament reconstruction was performed in a second surgery.

In total, 1.2 surgeries per patient (range, 1–4) were necessary to improve clinical outcome in the 70 patients in our study (57 patients needed only one operation, 12 patients had two operations, and one patient had four operations).

**Fig. 7:**
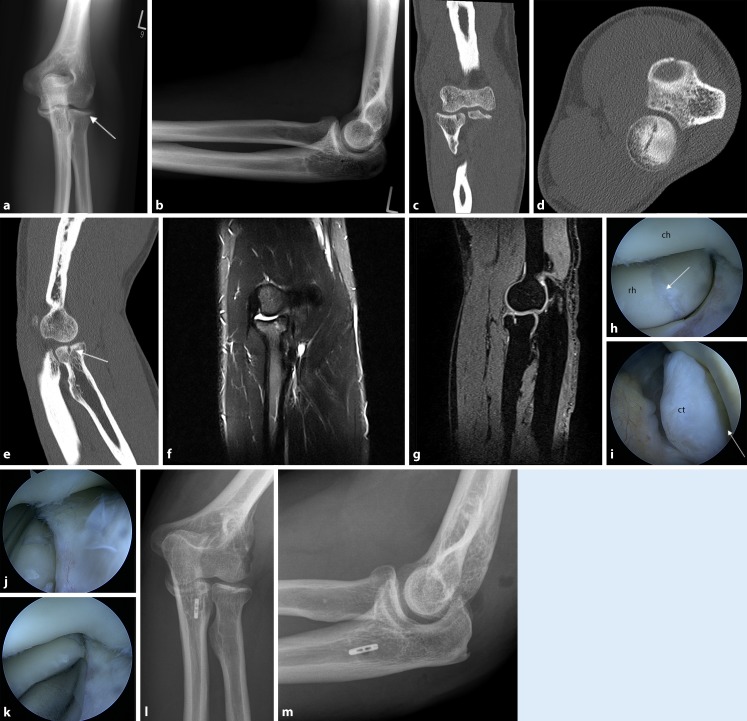
Case report: A 50-year-old patient with a radial head fracture (Mason I) after a fall had persistent symptoms after initial conservative treatment for more than 1 year. Radiography and computed tomography images show no step formation (*arrows*) at the radial head (**a**–**e**). Magnetic resonance imaging confirms the diagnosis of a Mason I fracture, but no soft tissue damage can be detected (**f**, **g**). The intraoperative results 1 year posttrauma show the former fracture (*arrow*) at the radial head (*rh*) and a gaping humeroradial joint gap (*arrow*) as an indication of lateral instability (*ch* capitulum humeri*,* *ct* coronoid tip; **h**–**k**). Postoperative radiograph after treatment with lateral ulnar collateral ligament repair (**l**, **m**)

When comparing the findings of clinical and arthroscopic stability testing, we found that 35 patients (50%) had a medial or lateral ligament instability in the preoperative clinical examination. In 94% of these cases, the instability was confirmed intraoperatively (25 cases of PLRI, five cases of ulnar instability, and three cases of combined instability). Finally, only 16 patients (45.7%) underwent ligament reconstruction:

- In all, 12 cases of lateral ulnar collateral ligament reconstruction (see case report in Fig. 7):
  - Two cases without arthroscopic treatment
  - Five cases in combination with an arthroscopic procedure
  - Four cases in a planned second surgery
  - One case due to recurrent instability
- Four cases of ulnar reconstruction:
  - Two cases without arthroscopic treatment
  - Two cases in a planned second surgery

In most cases, however, additional treatment (e.g., neutralizing the instabilities) besides arthroscopic arthrolysis was not necessary (52 cases, 74.3%).

The patients with nonstabilized fractures were nevertheless satisfied with the postoperative outcome, so that not all instabilities had clinical relevance in our series.

### Cartilage defect

Depending on the intraoperative findings, accompanying pathologies such as cartilage damage or hypertrophic plicae were addressed in the same surgery.

In only four cases (5.7%) were neither humero-ulnar nor humeroradial cartilage defects found. According to the Outerbridge classification [39], the humero-ulnar joint itself showed at least grade II or higher cartilage damage in approximately one out of six patients (15.7%). In the humeroradial joint (radial head and capitulum humeri), 90% of the patients already had grade II or higher cartilage lesions. Furthermore, 62.5% of all patients already had at least grade II–III cartilage lesions at the radial head itself, and every third patient (35.7%) had an advanced grade IV cartilage defect (Figs. 4b, c **and** 8).

**Fig. 8:**
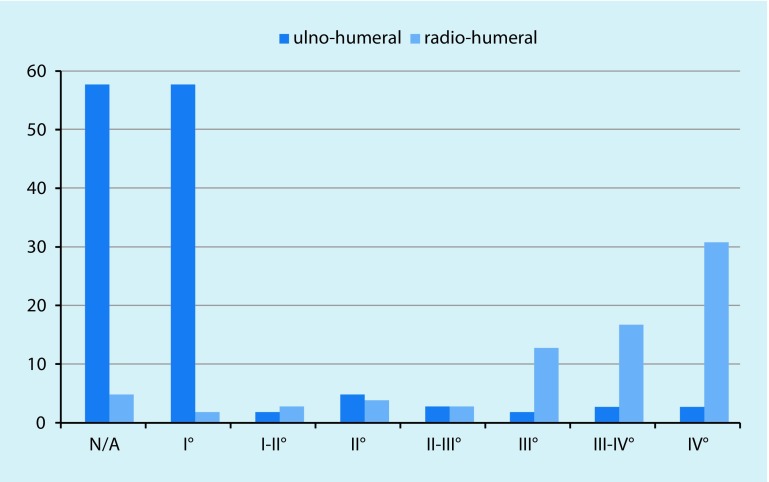
Cartilage damage according to joint section. A dramatic increase in cartilage damage occurs on the radial side of the elbow joint. *N/A* no cartilage damages

### Reoperation

In total, 13 patients needed 15 reoperations: two arthroscopic arthrolysis, four open arthrolysis, five lateral ligament reconstructions (one time due to recurrent instability), two ulnar ligament reconstructions, one hinged external fixator, one mobilization under anesthesia. However, nine of these operations were planned second interventions. Six complications were encountered in five patients including recurrent elbow stiffness and one recurrent lateral instability. Therefore, arthroscopic and open revision arthrolysis as well as one revision of a lateral ligament reconstruction had to be performed.

## Discussion

Radial head fractures tend to be underestimated by clinicians, in particular because of the lack of evidence of displacement on plain radiographs and the good results achieved with conservative treatment. However, these injuries may often be accompanied by a high degree of ligamentous and soft tissue as well as intra-articular cartilaginous damage, which cannot be displayed on radiographs [4]. Therefore, a thorough assessment of radial head fractures is necessary in order to determine the true severity and extent of these lesions and to minimize the risk of delayed surgical therapy. The correlation between an osseous lesion of the radial head and ligamentous injuries is well known. Itamura et al. described medial collateral ligament ruptures in 54%, lateral collateral ligament ruptures in 80%, and bilateral ruptures in 50% of all cases classified as Mason II and III fractures, while Mason IV fractures were excluded [19].

Hausmann et al. found a partial lesion of the interosseous membrane in nine of 14 patients with Mason I fractures using MRI [16]. Kaas et al. supported these findings, detecting accompanying injuries in Mason I–III fractures including lateral collateral ligament ruptures (*n* = 28) and humeroradial cartilage damage (*n* = 8) in 35 of 46 cases [22–24]. Whether concomitant injuries increase the necessity for surgical treatment remains controversial. Kaas et al. stated that most of the additional lesions in patients with radial head fractures are either not symptomatic or not of clinical importance. However, in their follow-up examination at least 12 months after trauma, flexion and extension deficits occurred in 45 and 43% of their patients, respectively. In addition, 13 of 40 patients described crepitus and one patient had locking due to a loose body. The authors claimed that no patient needed delayed surgery since symptoms were mild and without major restrictions [23]. In our case series, most of the patients were initially treated elsewhere and did not undergo MRI as they suffered from mainly Mason I and Mason II fractures (28/35 cases).

Conservative treatment for Mason I fractures is regarded the gold standard and yields good to excellent results with temporary immobilization for 5–7 days followed by early functional treatment [17, 43, 45]. However, Burkhart et al. reported on 16 patients with poor outcome after Mason I fractures due to fracture sequelae such as instability, loose bodies, and posttraumatic arthrosis. The trauma mechanism in radial head fractures is similar to that of elbow dislocations. An enhanced clinical and radiological evaluation (typically MRI) is required for type I Mason radial head fractures in order to detect soft tissue and ligamentous damage and to initiate adequate treatment [6]. MRI frequently reveals evidence of elbow dislocation in Mason type I fractures with severe soft tissue injuries. It remains unknown which of these soft tissue injuries might benefit from surgery. However, a study by Adolfsson et al. supports the assumption that the severity of soft tissue injuries correlates with complications. The authors reported on a cohort of patients who experience redislocation despite receiving proper conservative management for a simple elbow dislocation. During surgery they found complete avulsions of the medial and/or lateral collateral ligaments and muscle origins [1]. For this reason, the use of MRI to visualize the extent of soft tissue injuries is reasonable. This conclusion is supported by our study. In particular, subjective elbow instability, pre-arthrotic deformities, and restriction in elbow mobility frequently occur after Mason I and II fractures with a negative effect on the clinical outcome. Our approach always includes MRI in selected cases of insufficient clinical improvement during early follow-up even in non-dislocated Mason I fractures. Conservative treatment is aimed for, but concomitant injuries might call for surgery.

In the literature, conservative treatment is still regarded the method of choice for Mason II fractures. In a long-term follow-up study, Akesson et al. reported good to excellent results in about 82% of cases after conservative management of Mason II fractures. The rate of degenerative changes reached 82% for the injured and 21% for the uninjured elbow [2]. Surgical treatment led to similar clinical results (82% good to excellent) 22 years after open reduction and internal fixation (ORIF) in a study by Lindenhovius and colleagues. However, arthrosis was only detected in one of 16 cases in their study [28]. To date, there is no prospective study comparing ORIF with conservative treatment in Mason II fractures. Yoon et al. compared nonsurgical treatment with ORIF in partial articular radial head fractures, but the study was compromised by several biases and the conclusion is therefore limited [44]. The RAMBO trial was initiated in 2014 and aimed to address the question of whether Mason II fractures should be treated conservatively or surgically [5]. Unfortunately, no results from the trial have been published yet. A systematic review by Zwingmann et al. favored ORIF with screws in Mason type II fractures over osteosynthesis with pins or K‑wires and over conservative treatment because of better outcomes with the former approach. However, the selection of conservative studies included in the review, dated from 1981 to 1992, represents a possible bias [46].

Owing to the lower rate of degenerative changes, especially in young patients, we recommend surgery for Mason II fractures. However, the operative approach (screws, plate osteosynthesis, pins etc.) has to be assessed individually.

By contrast, in Mason type III and IV fractures, a surgical approach is the gold standard. The method to be followed, however, is still under discussion since ORIF, as the preferred therapy, and implantation of a radial head prosthesis or radial head replacement represent suitable treatment options [3, 20, 25, 26, 37, 40, 43]. All these strategies exhibit advantages and disadvantages and are mainly dependent on the expertise of the surgeon.

Our study involved only patients undergoing surgery for fracture sequelae such as instability, restricted range of motion (stiffness), or painful weight-bearing. Elbow stiffness described in the literature, which is significantly correlated with the duration of immobilization, is a serious complication after radial head fractures [8, 29, 30, 33–35, 41, 42]. This was also found in the present study. In most cases, a relevant restriction of elbow movement was one of the main reasons for delayed surgery after initial conservative treatment of non- or only slightly displaced radial head fractures. Regarding the soft tissue damage, some patients with Mason I radial head fractures likely had an injury mechanism similar to an elbow dislocation. Furthermore, the radial head is an important stabilizer against valgus stress in combination with the medial collateral ligament, which was confirmed by several biomechanical studies [12–14, 25, 36].

Soft tissue damage, especially in Mason I fractures, can lead to the classification being adapted after surgical intervention, thereby resulting in a Mason IV fracture.

In contrast to reports in the literature, in which cartilage lesions are described as either asymptomatic or clinically not relevant, our patients suffered remarkably from cartilage damage, which is mostly detected as crepitus during clinical examination. In our series, all complications and all pathologies represented surgical indications due to restricting symptoms. The difficulty of treating radiocapitellar arthritis especially in young patients is a frequent topic of discussion in the literature. Therefore, prevention of radiocapitellar arthritis seems logical. ORIF of Mason II fractures is a simple procedure with a very high rate of good results and low complication rates.

On the basis of our patient population, we cannot recommend primary surgical treatment for Mason I or Mason II fractures since we did not compare outcomes. Instead, appropriate and extensive diagnostics are necessary to detect concomitant injuries, which might influence the decision on whether the patient will benefit from surgery or not. Furthermore, continuous clinical examinations are highly recommended so as to change conservative treatment when required. We tend to recommend operative therapy for Mason II fractures in cases of concomitant lesions, since degenerative lesions mostly prevailed in our patient group with Mason II fractures.

Thus, measurements that possibly promote complications, such as lengthy immobilization or patients with suspicious injuries who refuse MRI, should be avoided. Although not applicable to all patients and all clinics, we recommend acquiring radiographs in two planes and performing MRI independent of the Masson classification of the fracture. The necessity for surgical treatment has to be evaluated individually in every patient with consideration of age, occupation, sports, and handedness [4, 7, 16, 19, 22, 23, 32].

Especially the treatment of intraoperatively detected instabilities by means of stabilization surgery (ligament repair or ligament reconstruction) should be assessed individually. In our study, not every arthroscopically detected instability needed surgical stabilization. Even without stabilization, adequate clinical results could be achieved, similar to the findings of Kaas and coworkers [22–24].

The lack of a classification system that combines bony and ligamentous lesions as well as the lack of prospective studies comparing surgical with conservative treatment does not allow for a general therapy algorithm. It is important to pay attention to so-called red flags like restriction of movement, unchanging high pain level, or simply unambiguous elbow instabilities in stress testing a few days after trauma.

## Practical conclusion

- Mason I fractures are associated with soft tissue injuries that might benefit from surgical treatment in select patients. Therefore, we tend to regard radial head fractures as osteoligamentary injuries.
- There is a higher probability of radial head fractures (type Mason II) resulting in posttraumatic osteoarthritis following conservative treatment compared with surgical treatment. Therefore, we recommend surgery to restore anatomy and address soft tissue damage if required.
- Accompanying injuries should be detected at an early stage and treated in a targeted, therapy-adapted manner. Nevertheless, it is still unclear which accompanying injuries are better addressed surgically and which not. In selected cases of insufficient clinical improvement at an early stage of follow-up, MRI should be performed independent of the Mason grade.
- The necessity for surgery must be made according to the MRI findings and the clinical symptoms. Because, independent of Mason grade, only MRI can reveal the full extent of bony and ligamentous injuries. An initially conservative treatment regimen can be changed in favor of an operative procedure in selected cases.
